# Toric IOL implantation in a patient with keratoconus and previous penetrating keratoplasty: a case report and review of literature

**DOI:** 10.1186/s12886-018-0895-y

**Published:** 2018-08-29

**Authors:** Karin Allard, Madeleine Zetterberg

**Affiliations:** 1 Department of Ophthalmology, Sahlgrenska University Hospital, Mölndal, Sweden; 2 Department of Clinical Neuroscience/Ophthalmology, Institute of Neuroscience and Physiology, The Sahlgrenska Academy at University of Gothenburg, SE-431 80 Gothenburg, Sweden

**Keywords:** Cataract, Keratoconus, Penetrating keratoplasty, Phacoemulsification, Toric intraocular lens

## Abstract

**Background:**

Cataract surgery in patients with keratoconus with or without previous penetrating keratoplasty (PKP) can be demanding due to difficulties in selecting the intraocular lens (IOL) and predicting the refractive outcome. We report a case of cataract surgery in a patient with keratoconus and previous PKP in one eye.

**Case presentation:**

A 71-year-old man with bilateral cataract and advanced bilateral keratoconus and previous PKP in the left eye. Preoperatively, best corrected visual acuity (BCVA) was 20/150, with − 5.75 sph − 9.75 cyl 72°, in the right eye and 20/40, with − 0.25 sph − 5.0 cyl 50°, in the left eye. The patient was subjected to phacoemulsification with implantation of a spherical IOL in the right eye and a toric IOL in the left eye. BCVA postoperatively was 20/80 with + 1.25 sph − 3 cyl 65° in the right eye and 20/25 with − 0.5 sph − 3.25 cyl 80° in the left eye.

**Conclusions:**

Correction of post-PKP astigmatism and cataract with phacoemulsification and implantation of a toric IOL can be an effective and safe choice. Predicting the refractive outcome in cataract surgery is difficult in patients with advanced keratoconus even when using non-toric IOLs, and the surgeon should be aware of different sources of biometric errors and the possible consequences.

## Background

Keratoconus is a common corneal ectatic disorder characterized by corneal thinning and protrusion of the cornea, resulting in irregular astigmatism and decreased vision [1]. The disease usually begins at puberty, progresses and stabilizes in the late 30’s [1]. Treatment is aimed at visual improvement and to prevent progress of the disease [1]. In the early stages of the disease the patients are treated with glasses or rigid contact lenses. Cross-linking with ultraviolet A (UVA) and riboflavin has emerged as an effective means of stabilizing the cornea and preventing progress of the disease. In more advanced stages, penetrating keratoplasty (PKP) may be needed [2, 3]. After PKP, corneal astigmatism - both regular and irregular - frequently occurs [4]. Despite a clear graft the post-operative astigmatism may limit the visual function. Full correction with glasses is not always possible because of high astigmatism and anisometropia. Contact lenses may be an option but can be difficult to fit because of irregularities of the cornea. Several surgical approaches have been tried to manage postoperative astigmatism but it still remains a challenge [5–7]. Cataract surgery in patients with keratoconus with or without previous PKP can be demanding due to difficulties in selecting the intraocular lens (IOL) and predicting the refractive outcome. When the patient has both post-PKP astigmatism and cataract, a few small studies have reported phacoemulsification with implantation of a toric IOL as a feasible treatment alternative [8–10]. With a single procedure both defects are corrected simultaneously. In this report, management of a patient with keratoconus and previous PKP in one eye and bilateral cataract is described. A toric IOL was implanted in the eye with post-PKP astigmatism and a spherical IOL was chosen in the other eye with advanced keratoconus. This report also provides a review of the current literature regarding implantation of a toric IOL after PKP.

## Case presentation

**Fig. 1:**
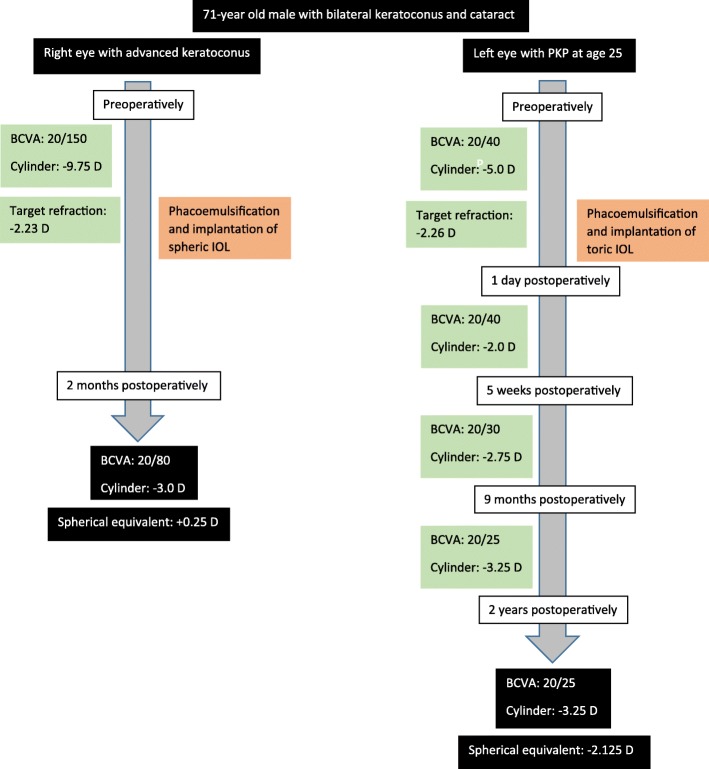
Timeline of the patient’s history, surgeries and follow-up

**Fig. 2:**
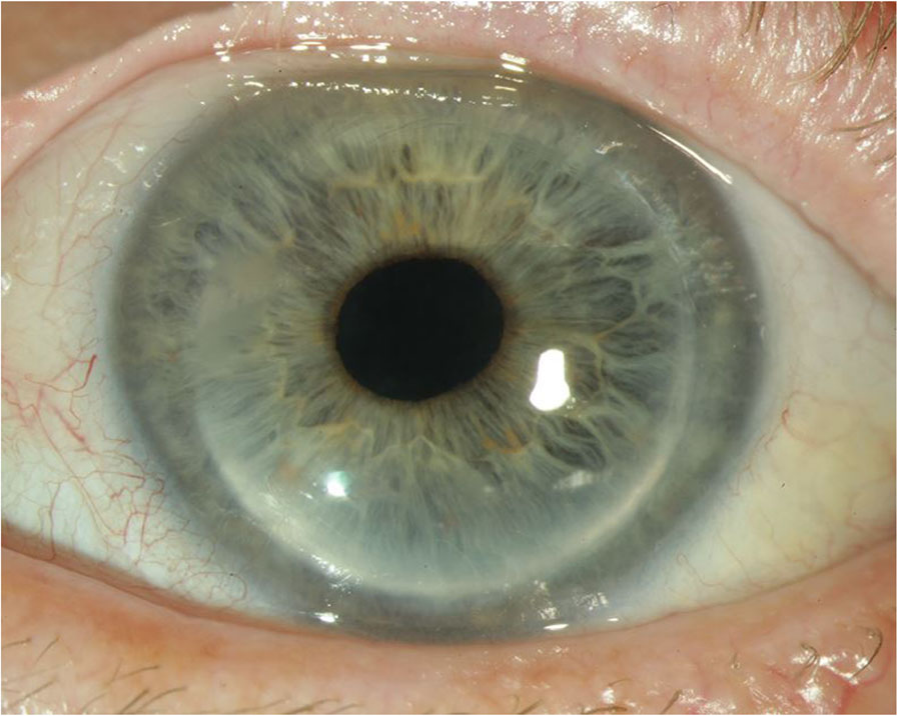
In the left eye PKP was performed when the patient was 25 years old

**Fig. 3:**
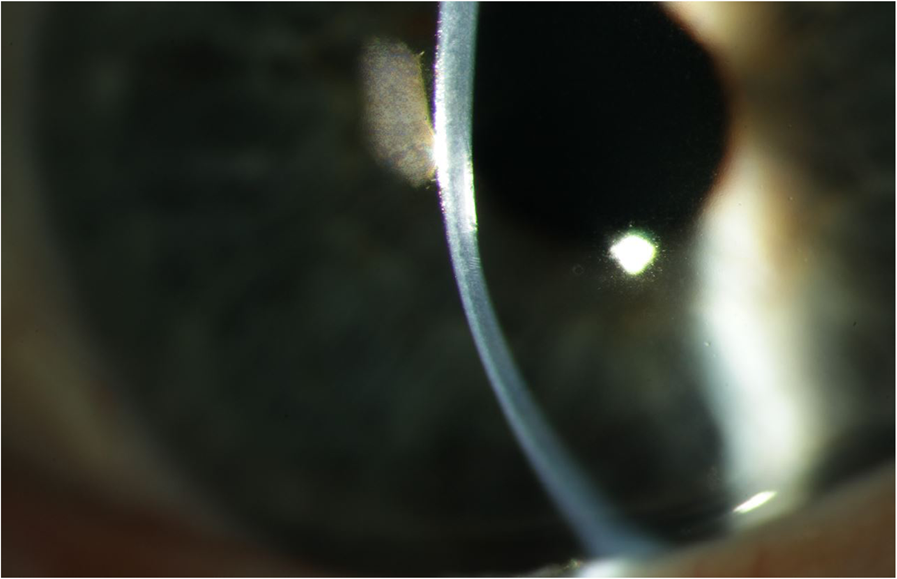
Vogt striae in the right eye with advanced keratoconus

A 71-year-old man with previously known keratoconus presented with bilateral cataract (Fig. 1). In the left eye, PKP had been performed when the patient was 25 years old because of keratoconus (Fig. 2). No surgery had been done in the right eye. Because of discomfort with contact lenses, the patient wore glasses both for near and far distance. The patient had a medical history of a transient ischemic attack and medicated with acetylsalicylic acid. The right eye presented with advanced keratoconus including Vogt striae (Fig. 3) in the cornea and moderate senile nuclear cataract but no other pathology. The left eye presented with a clear corneal graft and moderate senile nuclear cataract but no other pathology. First surgery was only planned in the left eye. After more than 1.5 years surgery was also performed in the right eye. Written informed consent was acquired from the patient.

### Left eye with previous PKP

**Fig. 4:**
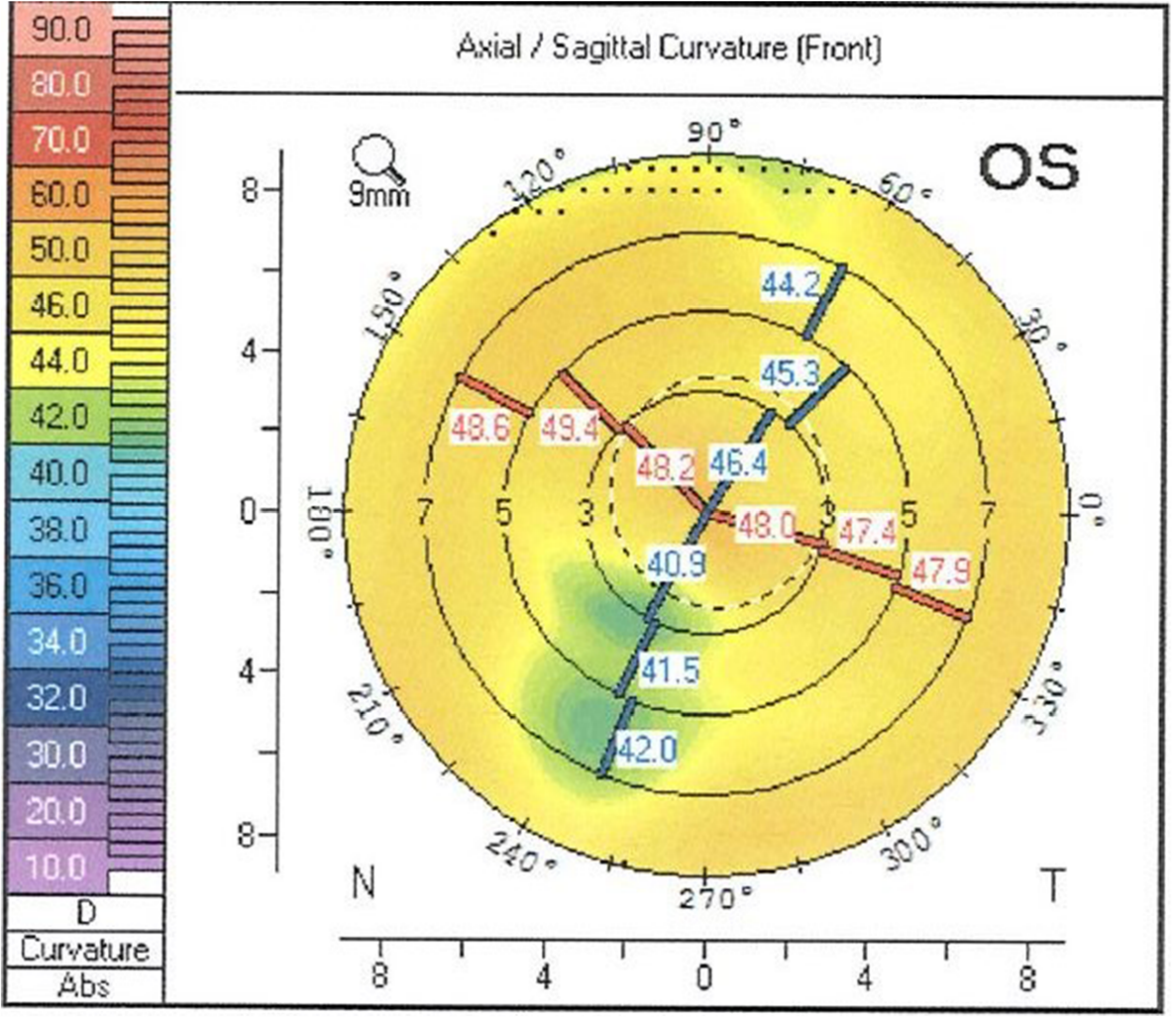
Scheimpflug imaging (Pentacam, Oculus, Germany) of the corneal front surface of the left eye with previous penetrating keratoplasty

**Fig. 5:**
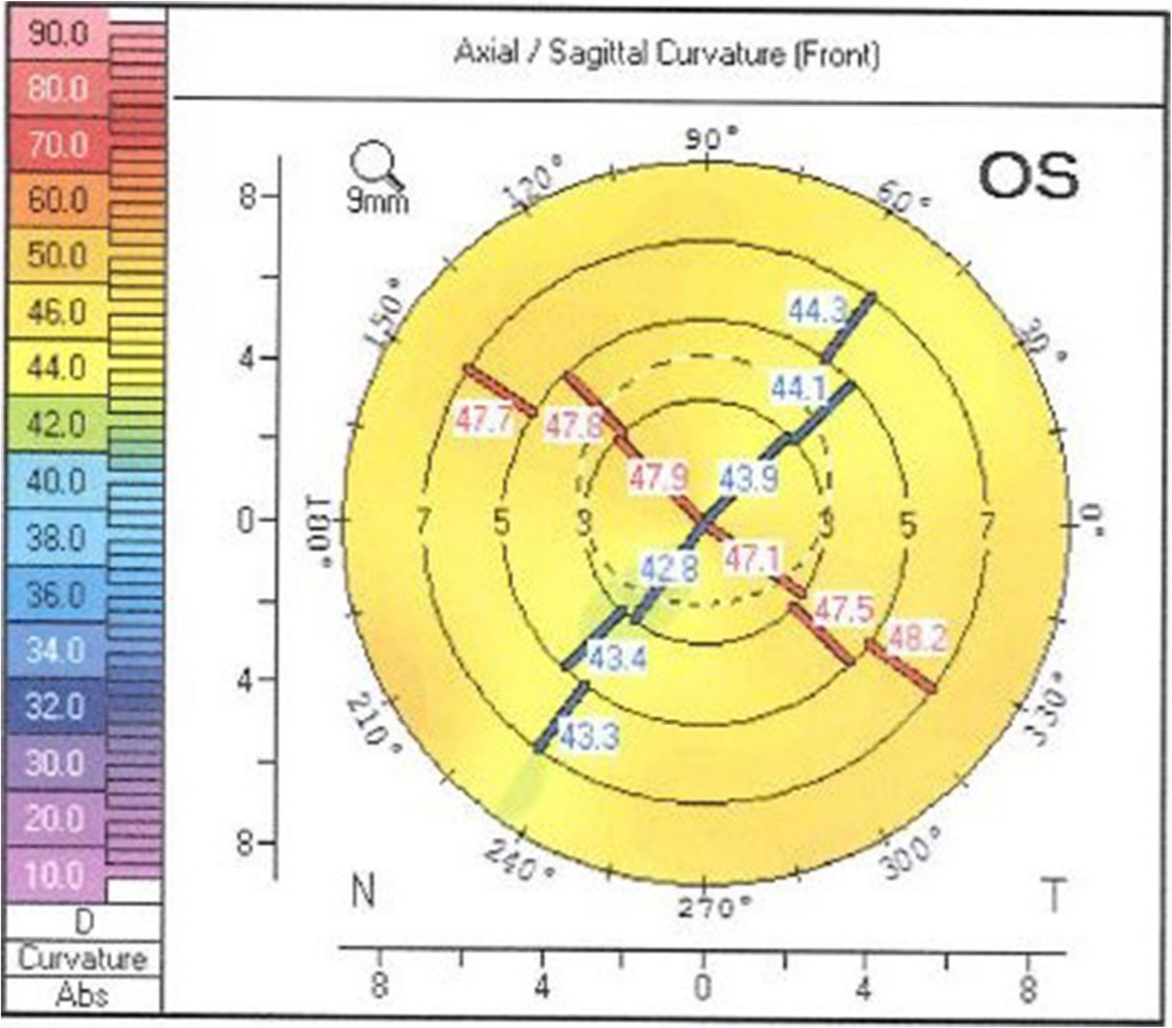
Scheimpflug imaging (Pentacam, Oculus, Germany) postoperatively of the corneal front surface of the left eye with previous penetrating keratoplasty

Preoperatively, best corrected visual acuity (BCVA) was 20/40, with − 0.25 sph − 5.0 cyl 50°. The cornea exhibited regular astigmatism (K1 44.5 D, K2 48.5 D, astigmatism 3.9 D) (Fig. 4) based on corneal tomography performed with Scheimpflug imaging (Pentacam, Oculus, Germany). The toric IOL AcrySof IQ Toric SN6AT8 (Alcon, USA), 22 D was implanted with target refraction − 2.26 D. The target refraction was chosen to match the more myopic right eye. Biometry was performed with the IOLMaster (Carl Zeiss Meditec, Germany) and Haigis formula was used. Preoperative marking of the toric IOL axis was performed with the patient in upright position to avoid misalignment due to cyclotorsion, using the RoboMarker (Surgilum, USA). Phacoemulsification and lens implantation were performed through a 2.2 mm limbal incision. One day postoperatively, BCVA was 20/40 with − 2.0 cyl 90°. Five weeks postoperatively BCVA was 20/30 with + 0.5 sph − 2.75 cyl 71°. Nine months postoperatively BCVA had improved to 20/25 with − 3.25 cyl 90° and the astigmatism was still regular (Fig. 5) based on corneal tomography performed with Scheimpflug imaging. Two years postoperatively BCVA was still 20/25 with − 0.5 sph − 3.25 cyl 80°. The spherical equivalent 2 years postoperatively only differed − 0.135 D from the intended target refraction. During all postoperatively controls the toric IOL only misaligned 1° (from 139° to 140°) from the implanted axis and the corneal graft remained clear. The patient was very satisfied with the visual result from day one postoperatively.

### Right eye with advanced keratoconus

**Fig. 6:**
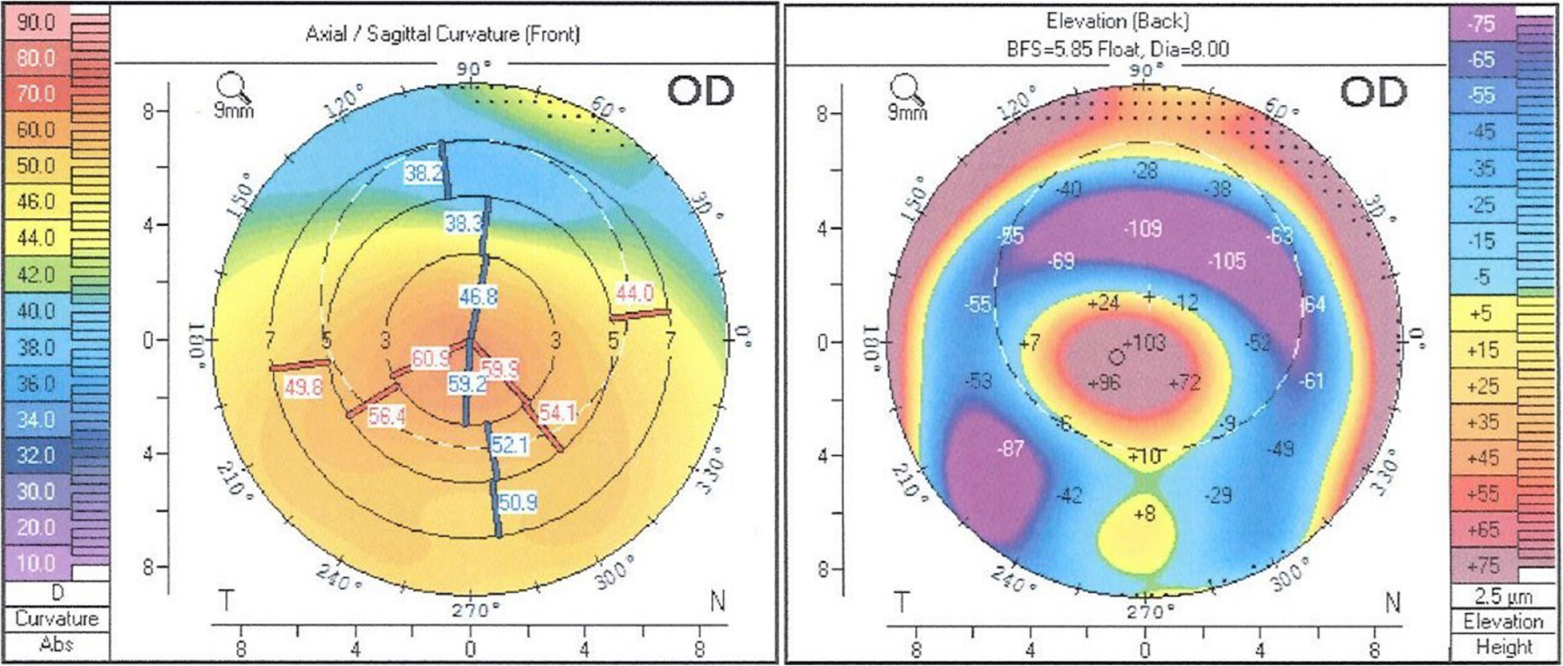
Scheimpflug imaging (Pentacam, Oculus, Germany) of the corneal front and back surface of the right eye with advanced keratoconus

Preoperatively, BCVA was 20/150, with − 5.75 sph − 9.75 cyl 72° and the cornea had irregular astigmatism (K1 53 D, K2 57.7 D, astigmatism 4.7 D) (Fig. 6) based on corneal tomography performed with Scheimpflug imaging. In the right eye, the astigmatism was judged as being too irregular for toric IOL implantation and cataract surgery was performed with the spherical IOL Acrysof Multipiece MN60MA (Alcon, USA), 5 D. Conventional biometry (IOLMaster) and Haigis formula was used to calculate the power of the spherical IOL. Two months postoperatively, BCVA was 20/80 with + 1.25 sph − 3 cyl 65°, spherical equivalent − 0.25. Target refraction prior to surgery was − 2.33 D, however the patient was pleased with the obtained result. He continues to wear glasses for far distance but does not require glasses for near distance.

## Discussion and conclusions

Our patient exhibited a decrease in astigmatism and increased BCVA after cataract extraction and implantation of a toric IOL in the left eye which had post-PKP astigmatism, with rotational stability of the IOL. The results remained stable during the follow-up time of 2 years. The patient was very pleased with the visual result even though we were not able to completely eliminate the corneal astigmatism (residual astigmatism of − 3.25 D). The residual astigmatism is somewhat surprising since preoperative Scheimpflug imaging showed regular astigmatism. However, it should be remembered that this eye had been subjected to PKP, which may make the outcome more difficult to predict.

The good results in our case is in agreement with previously reported results [8–10]. Only small case-reports and case-series have been performed so far, the largest with 22 eyes is a retrospective case-series [8]. A concern with toric IOLs is rotational stability. Postoperative rotation of 10° reduces astigmatic correction by 30% and a rotation of 20° reduces astigmatic correction by 60% [11]. Our case and other studies [8–10] show stability of the toric IOL after implantation in a post-PKP eye. Another concern in cataract surgery is endothelial cell loss. In eyes with previous PKP the loss of endothelial cells during cataract surgery is even more pronounced [12]. For the present case we did not count endothelial cells preoperatively nor postoperatively since this is not standard procedure in our clinic but the patient’s corneal graft showed no signs of decompensation even after 2 years follow-up. In our patient, post-PKP astigmatism was regular, but this is often not the case. One study found that 72% of patients had irregular astigmatism 12 months after PKP [13]. Irregular astigmatism is not suitable for correction with toric IOLs [10]. Therefore, toric IOLs should be reserved for those PKP-patients who have predominantly regular astigmatism.

In the present case, the second eye - which had advanced keratoconus but no previous PKP - astigmatism decreased and BCVA increased after cataract extraction and implantation of a spherical IOL. We believe that this was due to the surgical incisions. The follow-up time of this eye was only 2 months however. The spherical equivalent at follow-up differed − 2.08 D from the intended target refraction but the patient was very pleased with the outcome. Performing cataract surgery in patients with keratoconus needs planning and careful considerations. Accurate keratometry measurements are difficult which results in inaccurate corneal power estimates and difficulties in selecting the power of the IOL [14–16]. In eyes with keratoconus it cannot be assumed that the measured K-value is equal to the K-value at the visual axis nor that the effect of the measurement error is uniform for all keratometric values [16]. Typically, conventional biometry (IOLMaster) will overestimate the corneal power and underestimate the IOL power in eyes with keratoconus, resulting in postoperative hyperopia [16]. In eyes with mild (Kmax< 48 D) and moderate (Kmax = 48–55 D) keratoconus these effects are usually small and using K-values from the IOLMaster results in acceptable refractive outcomes [16].

In conclusion, correction of post-PKP astigmatism and cataract with phacoemulsification and implantation of a toric IOL can be an effective and safe choice. Prospective studies with larger cohorts are required to further investigate which patients may benefit from treatment with toric IOLs and to conclude on the efficacy. Predicting the refractive outcome in cataract surgery is difficult in patients with keratoconus even when using non-toric IOLs, and the surgeon should be aware of different sources of biometric errors and the possible consequences.

## Abbreviations

BCVA: Best corrected visual acuity
IOL: Intraocular lens
PKP: Penetrating keratoplasty
